# Effectiveness of Sofosbuvir and Daclatasvir in treatment of Hepatitis-C: An experience of tertiary care hospital in Karachi

**DOI:** 10.12669/pjms.37.7.4627

**Published:** 2021

**Authors:** Nazish Butt, Anoshia, Muhammad Ali Khan, Ali Akbar

**Affiliations:** 1 Dr. Nazish Butt, MBBS, FCPS. Assistant Professor, Head Department of Gastroenterology, Department of Gastroenterology, Jinnah Postgraduate Medical Center, Karachi, Pakistan; 2 Dr. Anoshia, MBBS, FCPS. Consultant Gastroenterologist, Department of Gastroenterology, Jinnah Postgraduate Medical Center, Karachi, Pakistan; 3 Dr. Muhammad Ali Khan, MBBS. Department of Gastroenterology, Jinnah Postgraduate Medical Center, Karachi, Pakistan; 4 Dr. Ali Akbar, MBBS, FCPS. Consultant Gastroenterologist, Department of Gastroenterology, Jinnah Postgraduate Medical Center, Karachi, Pakistan

**Keywords:** Hepatitis C, Daclatasvir, Sofosbuvir, Ribavirin

## Abstract

**Objective:**

To assess the effectiveness of Sofosbuvir (SOF) and Daclatasvir (DCV) in patients with chronic hepatitis C (CHC), compensated cirrhosis (CC) and decompensated cirrhosis (DCLD) either treatment naïve or experienced.

**Methods:**

This was a prospective, observational study, conducted from January 2017 to December 2018 at Jinnah Postgraduate Medical Centre, Karachi. All patients above 12 years of age with detectable HCV RNA PCR were included. Patients were divided into three groups: CHC, CC and DCLD. SOF and DCV for 12 or 24 weeks were given. Ribavirin (RBV) was given to treatment experienced and cirrhotic patients. Primary outcome was End of Treatment Response (ETR) and secondary outcome was Sustained Virological Response (SVR) at post treatment week 12 or 24.

**Results:**

Total 300 patients with mean age of 40.49 ± 13.86 were enrolled. Majority were females 174 (58%). CHC were 200 (66.6%) while cirrhotic were 100 (33.4%). Treatment naïve patients were 267 (89%) and 33 (11%) patients were experienced. Most common genotype was 3 (83%). ETR was achieved in 292 (97.33%) and SVR in 265 (88.33%) patients respectively.

**Conclusion:**

SOF plus DCV with or without RBV is a highly effective treatment for chronic HCV and is still used in many centers of Pakistan. This regimen has excellent results for GT-3. The outcomes are mainly influenced by the presence or absence of cirrhosis.

## INTRODUCTION

Hepatitis C virus (HCV) has an estimated prevalence of 115 million worldwide.^1^ It can cause acute or chronic hepatitis that leads to cirrhosis in 10-20% and hepatocellular carcinoma (HCC) in 1-5% of the patients.^2^ Even today HCV is the leading cause of decompensated liver disease (DCLD) in the developed world.

HCV has six genotypes 1-6. The most prevalent is genotype-1 (GT-1), with an estimated prevalence of 46% followed by Genotype-3 (GT-3) globally.^3^ GT-3 has a higher incidence in Asian countries especially Pakistan and India whereas GT-1 makes up only a fraction of the total cases of HCV here. Historically genotype 1 (GT-1) had the best treatment outcomes, while Genotype-3 (GT-3) had the worst outcomes. All of this changed after the approval of Direct Acting Antivirals (DAAs). Initially Sofosbuvir (SOF), NS5B inhibitor was used in conjugation with other drugs in various combinations for specific genotypes with superb results.

Daclatasvir (DCV), a NS5A inhibitor was first approved in 2015 by the Food and Drug Authority to be used in combination with SOF for treatment of HCV genotypes 1&3. Its introduction was a major step towards a pan-genotypic regimen. Its efficacy for treating genotype 4 remains unproven to this day but is still used in many government setups because of limited options and affordability issues. There is ample data available on the efficacy and safety of the SOF+DCV regimen in treating HCV in the west. However, focused researches from Pakistan in this matter are lacking. With such a high rate of viremia^4^ in cirrhotics and non cirrhotics, insights into treatment options for HCV in Pakistan are much needed. Through this study we hope to enlighten our society about just one such option.

## METHODS

This was a prospective observational study conducted at Department of Gastroenterology, Jinnah Postgraduate Medical Centre Karachi after the approval of Institutional Review Board (No.F.2-81/2019-GENL/9025/JPMC, dated 16-01-2019) from January 2019 to December 2020. Consecutive, non-probability sampling was done.

### Inclusion criteria

All patients of either gender with ages more than 12 years whose HCV RNA detected through Polymerase Chain Reaction (PCR) with a lower limit of 15 IU/ml. Liver disease was staged into three categories.

### Chronic Hepatitis C (CHC)

Patients with active viral replication with detectable PCR, without any clinical, laboratorial or radiological evidence of cirrhosis.

### Compensated Cirrhosis (CC)

Patients with active viral replication with evidence of cirrhosis on ultrasound or transient elastography (TE) with liver stiffness measurement score of ≥ 13 KPa or having endoscopic evidence of varices or portal hypertension were included in this category.

### Decompensated Cirrhosis (DCLD)

Patients with active viral replication and any previous or recent evidence of ascites, porto-systemic encephalopathy or variceal bleed along with sonological finding of shrunken liver with irregular margins and/or splenomegaly.

### Exclusion criteria:

Patients with uncontrolled hypertension, diabetes mellitus, unstable heart failure, eGFR<30ml/min, hemoglobin level of <8.5g/dL, hepatocellular carcinoma and active tuberculosis were excluded.

Data was collected from each patient included basic socio-demographic information such as gender, occupation, history of co-morbidities such as Diabetes, Hepatitis B, signs and symptoms such as dyspepsia, jaundice, weight loss, fatigue, porto-systemic encephalopathy, upper gastrointestinal bleeding and ascites.

Baseline investigations including complete blood count, liver function tests and abdominal ultrasound. The genotype of each patient was categorized through HCV RNA PCR.SOF was given 400 mg/day, DCV was given 60 mg/day and RBV was given 1000 mg/day if weight was < 75 KGs and 1200 mg/day if > 75 KGs. In DCLD group RBV was started with 600 mg/day and was titrated up accordingly. Four distinct treatment protocols were instituted based on the stage of liver disease and history of previous treatment or lack thereof. The protocols are as follows:

1. CHC (naïve) = SOF+DCV = 12 weeks.
2. CHC (experienced) = SOD+DCV+RBV = 12 weeks
3. CC (naïve) = SOD+DCV = 24 weeks.
4. CC (experienced) and DCLD (naïve/experienced) = SOF+DCV+RBV = 24 weeks.

The goal of therapy and primary outcome was complete elimination of virus at the end of treatment (ETR). ETR was defined by HCV RNA level below level of quantification at the end of treatment week 12 or 24. Secondary outcome was Sustained Virological Response (SVR) i.e. HCV RNA level below level of quantification at post treatment week 12 and 24. Both primary and secondary outcomes were divided into CHC, CC and DCLD subgroups. Subgroup analysis was also done.

The data was analyzed using SPSS version 22. Descriptive analysis was performed by calculating frequencies and percentages for categorical variables (like age, gender, HCV genotypes) and means & standard deviations for continuous variables. Inferential analysis was performed by applying chi-square test for nominal data for one or two groups whereas one-way ANOVA was used for more than two unrelated groups. The significance level was set at 0.05.

## Results

**Fig.1:**
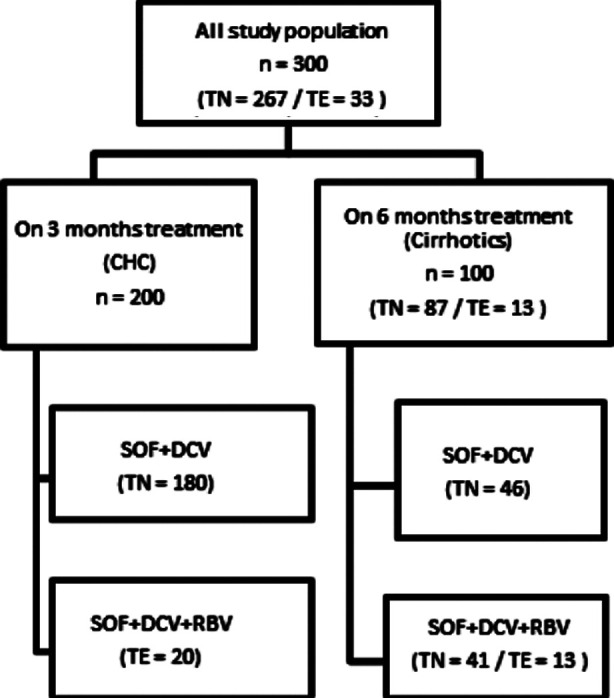
Flow chart of study patients. TN=Treatment Naive, TE=Treatment Experience, SOF=Sofosbuvir, DCV= Daclatascir, RBV=Ribavirin, CHC=Chronic Hepatitis C

Three hundred patients were inducted into the study (Fig.1). Two thirds of the patients had CHC only while one third of study population was cirrhotic. Mean age at presentation was 40.49 ± 13.86 years. Males were 42% and females were 58%. The demographics are shown in Table-I.

**Table I:** Baseline characteristics, demographics and lab parameters.

	*N=300*
***Baseline characteristics and Demographics***
Age (mean)	40.49 ± 13.86 years
***Gender***	
Male	126 (42%)
Female	174 (58%)
***Lab parameters***	
Hemoglobin (g/dL)	12.00 ± 2.16
Mean Corpuscular Volume (fL)	81.22 ± 8.22
Platelet Count (cmm)	220.43 ± 101.66
Total Leukocyte Count (cmm)	7.04 ± 2.44
Prothrombin Time (sec)	12.09 ± 5.52
International Normalized ratio	1.09 ± 0.33
Total Bilirubin	0.94 ± 0.83
Direct Bilirubin	0.69 ± 3.79
Alanine Aminotransferase	65.57 ± 115.85
Alkaline phosphate	197.35 ± 96.82
Gamma-Glutamyl Transpeptidase (units/L)	42.60 ± 61.07
Albumin	3.66 ± 0.71
Urea	23.91 ± 13.38
Creatinine	0.80 ± 0.38

Numerical data presented in mean ±standard deviation, Categorical presented in N (%).

Baseline characteristics and lab investigations are summarized in Table-I showing elevated Alanine Aminotransferase (ALT) and Gamma-glutamyl Transpeptidase (GGT) levels. Both were indicative of active viral replication and liver damage.

**Fig.2:**
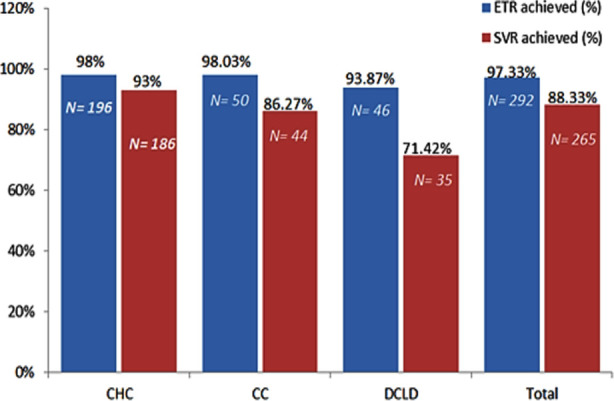
Primary and secondary outcomes.

In 249 (83.0%) GT-3 was found to be the culprit for all three stages of liver disease. Prevalence of HCV genotypes is summarized in Table-II. Two hundred and sixty-seven (89%) patients were treatment naive. Stages of liver disease and treatment status with response are summarized in Table-II.

**Table II:** HCV genotypes, liver disease stages & treatment status with type of regimen, duration and response.

			*N=300*
***HCV genotypes***			
Genotype 1			29 (9.7%)
Genotype 2			2 (0.7%)
Genotype 3			249 (83.0%)
Genotype 4			11 (3.7%)
Not Known			9 (3.0%)
***Liver Disease Stage***			
Chronic Hepatitis C (CHC)			200 (66.7%)
Compensated cirrhosis (CC)			51 (17.0%)
Decompensated CLD (DCLD)			49 (16.3%)
Genotype	GT-1	CHC	22 (75.9%)
		Compensated Cirrhosis	4 (13.8%)
		DCLD	3 (10.3%)
	GT-2	CHC	1 (50.0%)
		Compensated Cirrhosis	1 (50.0%)
	GT-3	CHC	163 (65.5%)
		Compensated Cirrhosis	43 (17.3%)
		DCLD	43 (17.3%)
	GT-4	CHC	8 (72.7%)
		Compensated Cirrhosis	2 (18.2%)
		DCLD	1 (9.1%)
	Unknown	CHC	6 (66.7)
		Compensated Cirrhosis	1 (11.1)
		DCLD	2 (22.2)
***Treatment Status***			
Treatment Naïve (TN)			267 (89.0%)
Treatment Experience (TE)			33 (11.0%)
***TE Previous Regimen***			
Conventional INF			3 (9.1%)
RBV + PEG			1 (3.0%)
RBV + SOF			26 (78.8%)
RBV + PEG + SOF			3 (9.1%)
***TE Duration***			
3 months			21 (7%)
6 months			12 (4%)
***TE Response Type***			
Non responder			7 (21.2%)
Relapse			23 (69.7%)
Defaulter			3 (9.1%)

ETR was achieved in 27 (93.1%) with genotype 1, 2 (100%) in genotype 2, 246 (99.2%) in genotype 3 and 9 (81.8%) in genotype 4 patients and there was significant difference P-value < 0.05. SVR was achieved in 20 (74.1%) in genotype 1, 2 (100%) in genotype 2, 229 (97.9%) in genotype 3 and 6 (54.5%) in genotype 4 and there was significant difference P-value < 0.05 as shown in Table-III.

**Table III:** Subgroup analysis.

		*ETR*		*P-value*	*SVR*		*P-value*
	
		*Achieved*	*Not achieved*		*Achieved*	*Not achieved*	
Genotype	Genotype 1	27 (93.1%)	2 (6.9%)	0.001*	20 (74.1%)	7 (25.9%)	0.001*
	Genotype 2	2 (100.0%)	0		2 (100.0%)	0	
	Genotype 3	246 (99.2%)	2 (0.8%)		229 (97.9%)	5 (2.1%)	
	Genotype 4	9 (81.8%)	2 (18.2%)		6 (54.5%)	5 (45.5%)	
Diagnosis	CHC	191 (98.5%)	3 (1.5%)	0.486	180 (95.7%)	8 (4.3%)	0.084
	Compensated Cirrhosis	49 (98.0%)	1 (2.0%)		45 (91.8%)	4 (8.2%)	
	DCLD	44 (95.7%)	2 (4.3%)		32 (86.5%)	5 (13.5%)	

* P-value < 0.05 was statistically significant.

When compared with different disease stages, it was found that ETR was achieved in (98%) patient with CHC, in (98%) CC and in (95.8%) DCLD patients and there was no significant difference. While SVR was achieved in (95%) with CHC, (88%) CC and (92%) with DCLD and there was no significant difference P-value > 0.05 (Table-III).

## DISCUSSION

Chronic HCV infection is mostly asymptomatic,^5^ until it is diagnosed incidentally. Pakistan is an intermediate endemecity region with respect to HCV,^6^ where most patients acquire the infection during adolescence or young adulthood. Varied presentations with respect to age and liver stage disease were seen in this study.

The mean age of CHC patients, compensated cirrhotics and decompensated cirrhotics were 36.91±13.02, 44.64±13.80 and 50.55±10.90 years respectively. Younger age was associated with markedly less severe disease, but cases of CHC with minimal liver inflammation or disease activity in older patients weren’t uncommon.

Male to female ratio was 1:1.38. This bore no statistical significance. Two thirds of the patients had CHC only. These were mostly young patients, but not always as the oldest case in this group was a 70-year-old man.

Significant elevation of ALT and GGT levels was seen in all three stages of liver disease with approximately equal median values. This persistent inflammation and enzyme elevation across all stages of liver disease irrespective of age or gender demonstrates chronic HCV’s deadly potential to cause cirrhosis or hepatocellular carcinoma.^7^

HCV GT-3 is the second most prevalent genotype worldwide, but the prevalence of GT-3 in this study was highest i.e., 83%. Previous data shows that GT-4 was very rare with detection rates of less than 0.5% in the subcontinent^8^, however its prevalence in this study was 3.7%. Perhaps it was under diagnosed previously or maybe it is emerging as a new cause of chronic HCV now. Certainly, this is an area for further insight and research.

The fact that 89% of the patients were treatment naïve and were just recently diagnosed, underlies the importance of improving screening protocols if HCV is to be eradicated in the developing world.^9^

A crucial factor for HCV eradication is maintaining follow up of the previously treated patients. Those treated with interferon (INF) in the past and with DAAs more recently are prone to develop relapses, GT-3 is particularly notorious for this. Severe disease (presence of cirrhosis), experience of Peg-INF or conventional INF, RBV and NS5A associated resistance associated substitutions (RASs) are predictors of relapse in HCV GT-3.^10^ These factors also contribute to non-response.

Of the 33 treated patients, 23 relapsed. Nearly all of them were treated with SOF+RBV previously. INF based regimen were linked with the highest rate of non-responders. The study did not calculate out of how many patients that took the SOF+RBV regimen relapses occurred. Previous data shows excellent SVRs achieved with this regimen and relapses to be minimal.^11^

As noted by Mangia A, et al. relapse rates were expected to decrease with newer DAAs such as DCV and Velpatasvir (VEL) even in severe liver disease. The ETRs and SVRs in this study are in part a representation of this very fact. This study did not use VEL, but our results with use of all of the four aforementioned treatment protocols were in line with previous data.^12^,^13^

In a study by Umar et al. SVR was achieved in 83%.^14^ In 2018, a study by Abozeid M et al. showed SVR achieved in 98%.^15^ Overall ETR in our study was achieved by 97.33% of the patients, however overall SVR rate was at 88.33%. This precipitous drop in undetectable levels of viral replication over time can be attributed to three main factors i.e. advanced liver disease requiring use of RIB (and six months of therapy), high prevalence of GT-3 and previous experience with INF based regimen.

Advanced liver disease is associated with poor outcomes (lower SVRs) when using SOF based regimen.^16^ Even after treatment these patients are at an increased risk of development of HCC and complications of cirrhosis.^17^ These worrisome features require constant surveillance even after clinical and laboratorial improvement.^6^,^18^

SVR rate in the CHC group was 93%, 86.27% in the CC group and 71.42% in the DCLD group. There were two genotypes i.e., 1&3 with significant numbers in this study. Traditionally GT-3 has been the most difficult to treat and maintain remission. Yet, new data with DCV demonstrates SVR rates of 94-97% in non-cirrhotic and 59-69% in cirrhotics for GT-3.^19^ Advanced liver disease and previous treatment experience in these studies, further validating our findings.

A review of the last 30 years showed that being treatment naïve and non-cirrhotic, SVR rates of nearly 100% were achievable with different DAA regimen; there is agreement in real world and clinical data regarding GT-3 but geographical differences in SVR rates persist.^20^

HCV GT-1 treated with SOF plus DCV based regimen has previously demonstrated SVR rates approaching 100%.^21^ While in a study by Cheema et al. 2019 SVR was achieved in 90.62%.^22^ The SVR rate for GT-1 in our study was approximately 74.1%. Seven patients with GT-1 did not achieve SVR; all of them belonged to either the CC or DCLD group.

GT-1 only made up a fraction (<10%) of total patients in this study; numbers were not enough to have a statistical significance. Therefore, it would be inappropriate to draw conclusions from this data alone. Little data that is available to us here suggests that outcomes in GT-1 and GT-3 are equally adversely affected by cirrhosis and treatment experience with INF based regimen. Surveillance protocols for both genotypes should be evenly stringent.

Use of RIB in cirrhosis and a treatment duration of six months positively affected SVR rates. However, this did not bore any statistical significance.

### Limitations of study:

The authors would like to point out the following deficiencies in this study:

1. Testing for IL28 B genotyping was not done.
2. RAS(s) were not analyzed at all.

## CONCLUSION

SOF plus DCV is highly effective treatment regimen for chronic HCV and is available in many centers of Pakistan free of cost. This regimen is pan genotypic with excellent results for GT-3, which is also the most prevalent genotype in Pakistan. The outcomes are mainly influenced by the degree of cirrhosis or lack there of.

### Authors’ Contribution:

**NB** conceived, reviewed and final approval of manuscript

**A, MAK, AA** designed, conducted data collection, manuscript writing and statistical analysis.

**A** is responsible and accountable of accuracy of the study..
